# Bone marrow mesenchymal stem cell exosomes-derived microRNA-216a-5p on locomotor performance, neuronal injury, and microglia inflammation in spinal cord injury

**DOI:** 10.3389/fcell.2023.1227440

**Published:** 2023-09-12

**Authors:** Hao Xue, Bo Ran, Jie Li, Guorui Wang, Baolin Chen, Honggang Mao

**Affiliations:** ^1^ Department of Orthopaedic Medicine, Inner Mongolia Baogang Hospital, Baotou, Mongolia, China; ^2^ Orthopaedic Research, Inner Mongolia Medical University, Hohhot, Mongolia, China; ^3^ Trauma Orthopedics, Baotou Medical College, Baotou, Mongolia, China

**Keywords:** spinal cord injury, bone marrow mesenchymal stem cell exosomes, microRNA-216a-5p, locomotor performance, neuronal injury and inflammation

## Abstract

**Background:** MicroRNA-216a-5p (miR-216a-5p) mediates inflammatory responses and neuronal injury to participate in the pathology of spinal cord injury (SCI). This study intended to explore the engagement of bone marrow mesenchymal stem cell exosomes (BMSC-Exo)-derived miR-216a-5p in locomotor performance, neuronal injury, and microglia-mediated inflammation in SCI rats.

**Methods:** Rat BMSC or BMSC-Exo was injected into SCI rats. GW4869 treatment was adopted to suppress the exosome secretion from BMSC. Subsequently, miR-216a-5p-overexpressed BMSC-Exo (BMSC-miR-Exo) or negative-control-overexpressed BMSC-Exo (BMSC-NC-Exo) were injected into SCI rats.

**Results:** The injection of BMSC or BMSC-Exo enhanced locomotor performance reflected by Basso, Beattie & Bresnahan score (*p* < 0.001), and neuronal viability reflected by NeuN^+^ cells (*p* < 0.01), but attenuated neuronal apoptosis reflected by TUNEL positive rate, cleaved-caspase-3 expression, and B-cell leukemia/lymphoma-2 expression (*p* < 0.05). Additionally, the injection of BMSC or BMSC-Exo suppressed microglia M1 polarization-mediated inflammation reflected by IBA1^+^iNOS^+^ cells, tumor necrosis factor-α, interleukin (IL)-1β, and IL-6 (*p* < 0.01). Notably, the effect of BMSC on the above functions was retarded by the GW4869 treatment (most *p* < 0.05). Subsequently, the injection of BMSC-miR-Exo further improved locomotor performance (*p* < 0.05), while inhibiting neuronal apoptosis (*p* < 0.05) and microglia M1 polarization-mediated inflammation (*p* < 0.05) compared to BMSC-NC-Exo. Interestingly, the injection of BMSC-miR-Exo reduced toll-like receptor 4 (TLR4) (*p* < 0.01), myeloid differentiation factor 88 (*p* < 0.05), and nuclear factor kappa B (NF-κB) (*p* < 0.05) expressions versus BMSC-NC-Exo.

**Conclusion:** BMSC-Exo-derived miR-216a-5p enhances functional recovery by attenuating neuronal injury and microglia-mediated inflammation in SCI, which may be attributable to its inhibition of the TLR4/NF-κB pathway.

## Introduction

Spinal cord injury (SCI) is one of the most disabling and destructive neurological disorders, which severely affects patients’ quality of life (Ahuja et al., 2017b). According to the Global Burden of Disease Study 2019, the incident and prevalent cases of SCI are 0.9 million and 20.6 million in 2019 (Ding et al., 2022). In general, SCI patients often have a partial or complete loss of sensory and locomotor performances below the plane of injury, showing a range of clinical symptoms such as abnormal muscle tone, urinary and defecation disorders, and limb paralysis (McDonald and Sadowsky, 2002; Eli et al., 2021). In terms of treatment, the main neuroprotective therapies for acute or early SCI include surgery, medication, and vasopressor therapy (Ahuja et al., 2017a). Unfortunately, there is no way to reverse damage to the spinal cord completely, and effective strategies that promote functional recovery from chronic SCI are still lacking (Venkatesh et al., 2019). Under these situations, it is necessary to explore potential treatment that improves the neurological function and locomotor performance of SCI.

Bone marrow mesenchymal stem cell exosomes (BMSC-Exo) have exhibited certain therapeutic effects on SCI (Liu W. Z. et al., 2021). For example, one previous study illustrates that BMSC-Exo attenuates apoptosis and promotes autophagy in neuronal cells of SCI rats (Gu et al., 2020). In addition, another study discovers that BMSC-Exo suppresses neuronal apoptosis and accelerates locomotor performance recovery by activating the Wnt/β-catenin pathway in SCI rats (Li et al., 2019). Furthermore, BMSC-Exo facilitates the phagocytosis of macrophages to clean myelin debris, thereby improving functional recovery after SCI (Sheng et al., 2021). These studies indicate that BMSC-Exo could act as a promising regimen for SCI. Notably, BMSC-Exo possesses abundant RNA, lipids, and proteins; these contents play an essential role in the process of intercellular material information transfer (Kalluri and LeBleu, 2020). Therefore, the underlying mechanism of BMSC-Exo in treating SCI deserves further investigation.

MicroRNA-216a-5p (miR-216a-5p) possesses the ability to regulate neuroinflammatory responses, neuronal injury, and neurogenesis in several neurological diseases, such as traumatic brain injury and Alzheimer’s disease (Xu et al., 2020; Xin Y. et al., 2021; Shao, 2021). For instance, one study indicates that miR-216a-5p attenuates neuroinflammatory response by inhibiting the high-mobility group box 1/nuclear factor kappa B (NF-κB) pathway (Shao, 2021). In addition, another study elucidates that increased miR-216a-5p in brain-derived neurotrophic factor-induced MSCs-Exo promotes neurogenesis after traumatic brain injury in PC12 cells (Xu et al., 2020). In terms of SCI, one study illustrates that miR-216a-5p is enriched in hypoxic BMSC-Exo and may potentially participate in the hypoxic BMSC-Exo-mediated microglial polarization (Liu et al., 2020). However, it should be mentioned that the previous study only discloses a possibility that BMSC-Exo may result in the increase of miR-216a-5p under hypoxic conditions, thus further helping to strengthen functional recovery after SCI (Liu et al., 2020). While the direct engagement of BMSC-Exo-derived miR-216a-5p in SCI is unknown and should be further investigated.

Accordingly, this research intended to study the direct influence of BMSC-Exo-derived miR-216a-5p on locomotor performance, neuronal injury, and inflammation in SCI rats.

## Methods

### Animals and ethics

The adult Sprague-Dawley (SD) rats (8 weeks old) from Shanghai SLAC laboratory animal (Shanghai, China) were housed in standard conditions (light-shade alternating cycle for 12 h, 24°C ± 2°C, and humidity of 50%–60%). All experimental and surgical procedures were permitted by the Animal Care and Use Committee and carried out according to the National Institutes of Health Guidelines.

### BMSCs culture, GW4869 treatment, and exosomes separation

Rats were euthanized with cervical dislocation after being anaesthetized by isoflurane inhalation, then femurs and tibias were isolated. The muscle attached to the bone was removed to expose the marrow cavity. Bone marrow was flushed by low-glucose Dulbecco’s modified eagle medium (DMEM; Gibco, United States). The cell suspension was then filtered, centrifuged, and plated in low-glucose DMEM containing 10% fetal bovine serum (FBS; Gibco, United States). The BMSCs were harvested after the adherent cells reached 80–90% confluence, and the BMSCs at 3rd passage were used for further experiments. The immunophenotypes of BMSCs were identified by flow cytometry after being stained with antibodies against CD29, CD44, CD90, CD105, CD34, and CD45 (BD, United States). The GW4869 (10 μM; Yeason, China) was adopted to suppress the exosomes secretion of BMSCs, and the isolation of BMSC exosomes (BMSC-Exo) was carried out in the presence of Exosome Isolation Kit (Yeasen, China) according to the kit’s protocol.

### BMSCs transfection

The BMSCs were transfected with negative control (NC) mimics or miR-216a-5p mimics (Genepharma, China) using Lipofectamine 3000 (Invitrogen, United States) per the kit’s procedures. After 72 h of transfection, BMSCs were cultured with medium containing exosome-free FBS (Gibco, United States) for an additional 24 h. Afterwards, the exosomes of transfected BMSCs were isolated (named BMSC-NC-Exo and BMSC-miR-Exo), and reverse transcription quantitative polymerase chain reaction (RT-qPCR) was completed for detecting the level of miR-216a-5p in BMSCs or BMSC-Exo.

### Establishment and grouping of SCI model

The SCI model was constructed as previously described (Li X. et al., 2020). Briefly, a laminectomy was performed to expose the spinal cord. Then, the spinal cord was contused with Infinite Horizon Impactor (PSI, United States). The injection of BMSCs or BMSC exosomes was carried out via tail intravenous after the surgery.

Firstly, 30 rats were separated into 5 groups (n = 6 per group): the Sham group, which received laminectomy only and injection of PBS; the SCI group, which received SCI surgery and injection of PBS; the BMSC group, which received SCI surgery and injection of BMSCs (5×10^6^ cells in 500 μL PBS); the BMSC-GW group, which received SCI surgery and injection of GW4869-treated BMSCs (5×10^6^ cells in 500 μL PBS); and the BMSC-Exo group, which received SCI surgery and injection of BMSC-Exo (200 μg exosomes in 500 μL PBS).

Then, additional 24 SCI rats were adopted to determine the engagement of miR-216a-5p. Briefly, rats were separated into 4 groups (n = 6 per group) after SCI surgery. The rats in the SCI group were given injection of PBS, and the rats in the BMSC-Exo, the BMSC-NC-Exo, and the BMSC-miR-Exo groups were given injection of BMSC-Exo, BMSC-NC-Exo, or BMSC-miR-Exo (200 μg exosomes in 500 μL PBS), respectively.

The Basso, Beattie & Bresnahan (BBB) locomotor score (Wang et al., 2020) were applied to quantify the locomotion recovery at 1, 3, 7, 14, 21, and 28 days after SCI surgery. All rats were euthanized at Day 28, and samples from spinal cord lesions were obtained for further assays.

### Hematoxylin-eosin (HE), TUNEL, and immunofluorescence (IF) staining

The tissues from the spinal cord lesions were fixed in 4% paraformaldehyde (Sangon, China), embedded in paraffin, and sliced into 4 μm thick sections. The HE and TUNEL staining were carried out in the presence of HE or TUNEL Staining kits (Yeason, China). For IF staining, sections were blocked with 10% BSA (Beyotime, China) and incubated with anti-NeuN, anti-IBA1, or anti-iNOS antibodies (1:100, Abcam, United States), followed by incubation of fluorescently labeled secondary antibodies (1:250, Abcam, United States) for 1 h at 37°C. The DAPI (Servicebio, China) staining was used to mark the nuclei. The antibody staining was specific.

### RT-qPCR

RT-qPCR was performed to assess miR-216a-5p level in BMSCs, BMSC exosomes, or spinal cord lesion tissues. In brief, the total RNA was isolated with TRIzol reagent (Invitrogen, United States).

RT-qPCR was finished using Bulge-Loop™ miRNA PCR Kit (Ribobio, China) per the supplier’s instructions. The primer sequences were listed (5′-3′): miR-216a-5p (forward: GGG​TAA​TCT​CAG​CTG​GCA​A, reverse: CAG​TGC​GTG​TCG​TGG​AGT), U6 (forward: GCT​TCG​GCA​GCA​CAT​ATA​CTA​A, reverse: CGA​ATT​TGC​GTG​TCA​TCC​TT).

## ELISA

The RIPA reagent (Beyotime, China) was used to lyse spinal cord lesion tissues. The supernatant was centrifuged (16,000 rpm, 10 min) and quantified with BCA kit (Beyotime, China). Then, the level of tumor necrosis factor (TNF)-α, interleukin (IL)-1β, and IL-6 was measured with ELISA kits (MLBIO, China) according to the supplier’s instructions.

### Western blot

The total protein of spinal cord lesion tissues was isolated with RIPA buffer and quantified with BCA kit. Then, the protein was separated with SDS-PAGE (Willget, China) and transferred to nitrocellulose membranes (Willget, China). After being blocked by 5% BSA (Beyotime, China), the membranes were incubated with primary antibodies (4°C, overnight) and secondary antibodies (37°C, 1.5 h), successively. The ECL kit (Willget, China) was used to visualize the blots. The antibodies used in this study were listed: Cleaved-caspase3 (1:500; Affinity, China), BCL2 (1:500; Affinity, China), toll-like receptor 4 (TLR4) (1:1000; Abcam, United States), myeloid differentiation factor 88 (myD88) (1:1000; Abcam, United States), p-p65 NF-κB (1:500; Affinity, China), p65 NF-κB (1:500; Affinity, China), β-ACTIN (1:2000; Affinity, China), and goat-anti-rabbit secondary antibody (1:5000; Affinity, China). The antibody staining was specific.

### Statistical analysis

GraphPad software 7.0 (GraphPad, United States) was used for statistical analysis. Among the sham, SCI, BMSC, BMSC-GW, and BMSC-Exo groups, 6 biological replicative samples were tested in each group, and 30 rats were tested in total. Among the SCI, BMSC-Exo, BMSC-NC-Exo, and BMSC-miR-Exo groups, 6 biological replicative samples were tested in each group, and 24 rats were tested in total. Regarding BMSC transfection and exosome isolation, 3 biological replicative samples were tested. Comparisons were accomplished with one-way ANOVA followed by Tukey’s *post hoc* test. *p* < 0.05 was considered statistically significant.

## Results

### BMSC-exo enhanced locomotor performance in SCI rats

**FIGURE 1:**
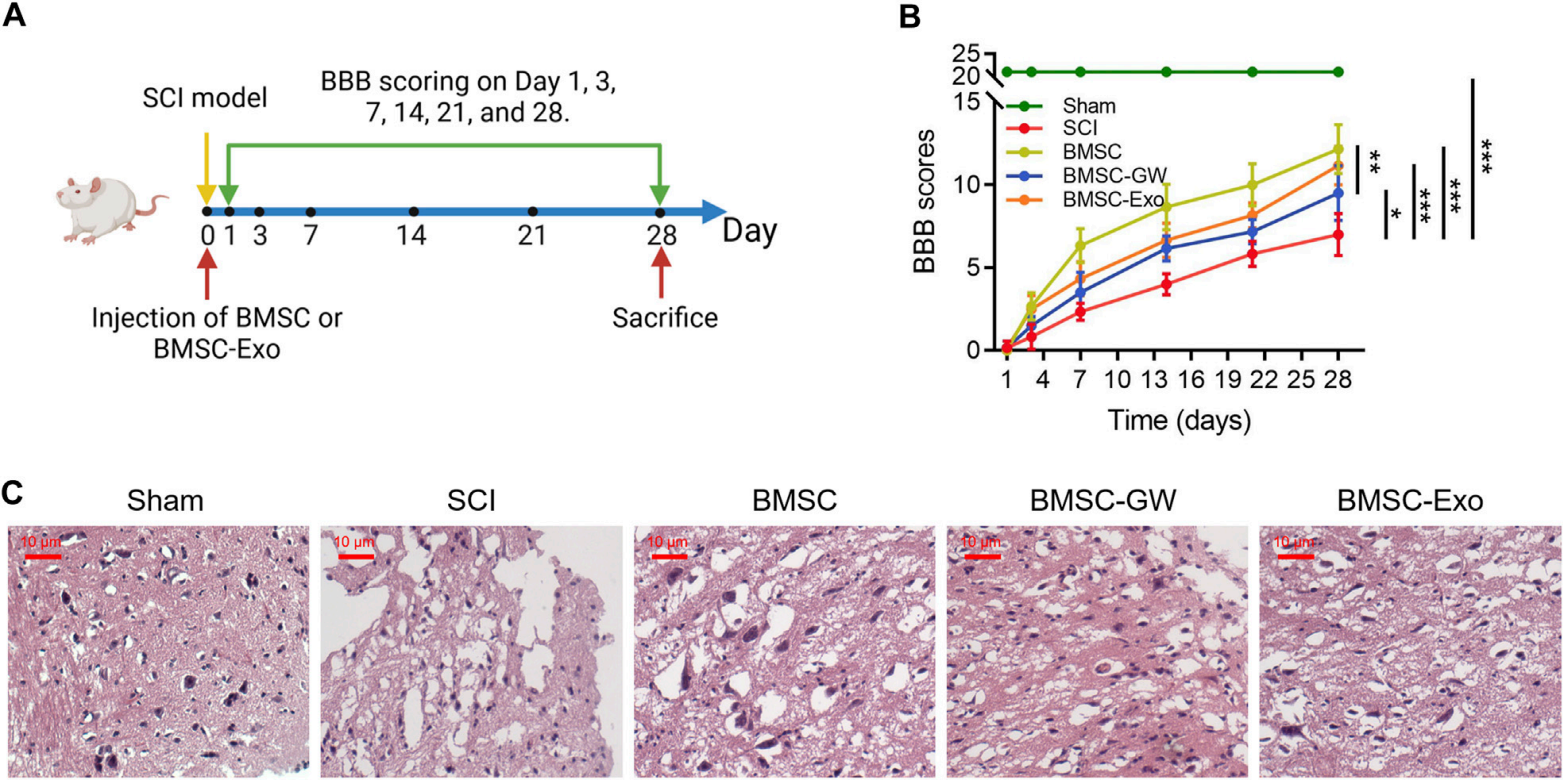
Effect of BMSC-Exo on locomotor performance in SCI rats. Experiment process **(A)**; comparison of BBB score **(B)** and representative images of HE staining **(C)**, among the sham, SCI, BMSC, BMSC-GW, and BMSC-Exo groups. Six biological replicative samples were tested in each group, and 30 rats were tested in total among the sham, SCI, BMSC, BMSC-GW, and BMSC-Exo groups.

Initially, the SCI model was established, then BMSC or BMSC-Exo was injected through the caudal vein. BBB score was evaluated on days 1, 3, 7, 14, 21, and 28. All rats were euthanized on day 28 (Figure 1A). Meanwhile, the BBB score on day 28 was higher in the BMSC group than in the SCI group (*p* < 0.001) and the BMSC-GW group (*p* < 0.01); in addition, the BBB score on day 28 was also higher in the BMSC-Exo group than in the SCI group (*p* < 0.001) (Figure 1B). Results of HE staining were shown in Figure 1C, which revealed that tissue injury and inflammation infiltration were reduced, while neuron numbers were increased in the BMSC group compared to the SCI group and BMSC-GW group. The same trend was found in the BMSC-Exo group versus the SCI group. The rat bone mesenchymal markers, including CD29^+^, CD44^+^, CD90^+^, CD105^+^, CD34^+^, and CD45^+^ were detected to ensure that the cell source came from rat bone mesenchymal. It turned out that the levels of CD29^+^, CD44^+^, CD90^+^, and CD105^+^ were 96.9%, 97.8%, 96.0%, and 98.1%, which were all over 95.0% and indicated that the cell source did come from rat bone mesenchymal. Meanwhile, the levels of CD34^+^ and CD45^+^ were 1.26% and 1.16%, which were less than 2.0% and indicated that the cell source did not come from other positions of rats (Supplementary Figure S1).

### BMSC-exo attenuated neuronal injury in SCI rats

**FIGURE 2:**
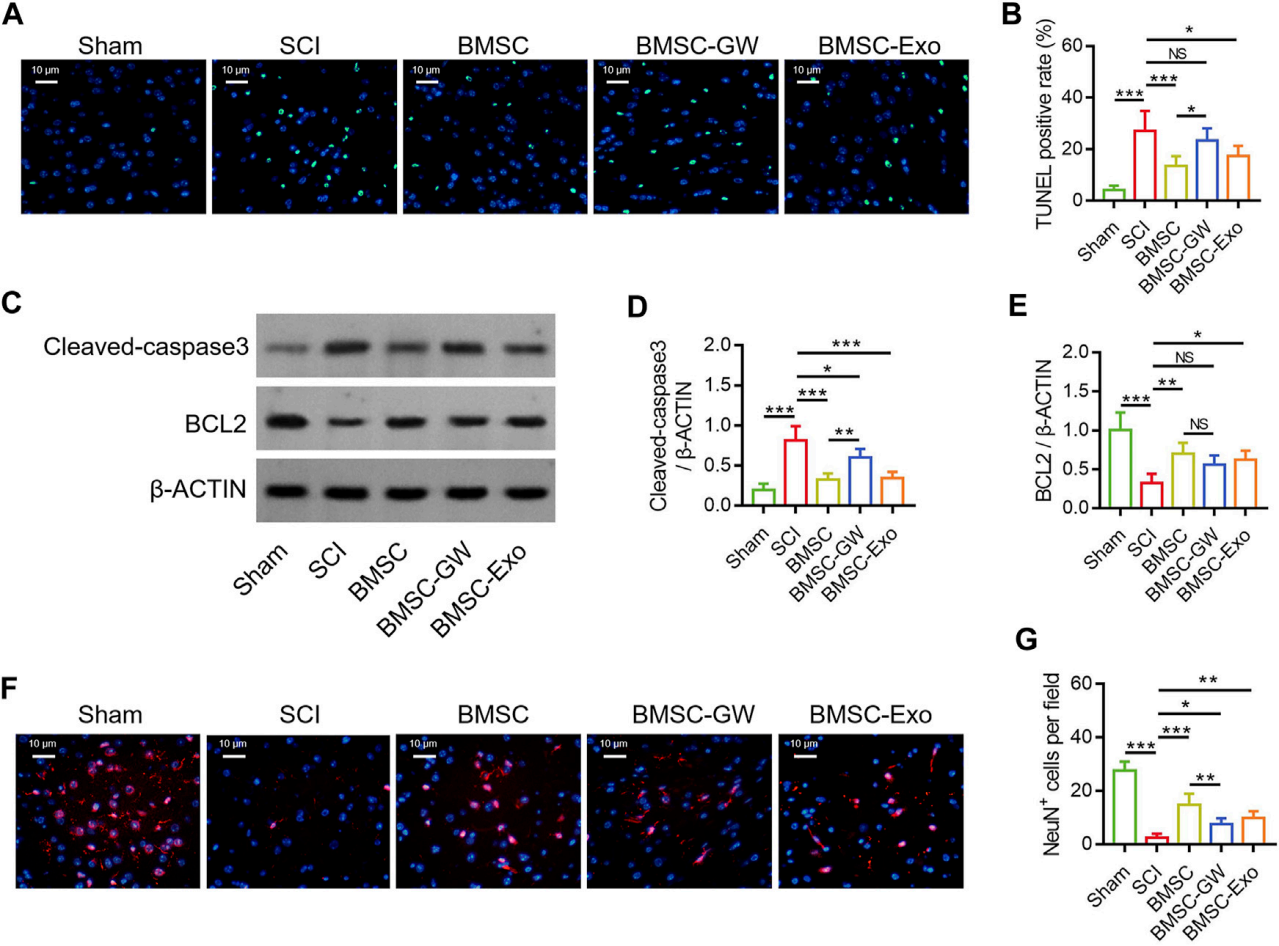
Effect of BMSC-Exo on neuronal injury in SCI rats. Representative images of cell apoptosis by TUNEL staining **(A)**, comparison of TUNEL positive rate **(B)**, detection of cleaved-caspase3 and BCL2 by Western blot **(C)**, comparison of cleaved-caspase3 **(D)** and BCL2 **(E)**, representative images of NeuN^+^ cells by IF staining **(F)**, and comparison of NeuN^+^ cells **(G)**, among the sham, SCI, BMSC, BMSC-GW, and BMSC-Exo groups. Six biological replicative samples were tested in each group, and 30 rats were tested in total among the sham, SCI, BMSC, BMSC-GW, and BMSC-Exo groups.

The TUNEL staining was conducted to detect cell apoptosis in SCI rats on day 28 (Figure 2A). TUNEL positive rate was diminished in the BMSC group versus the SCI group (*p* < 0.001) and the BMSC-GW group (*p* < 0.05). At the same time, TUNEL positive rate was also reduced in the BMSC-Exo group versus the SCI group (*p* < 0.05) (Figure 2B). Subsequently, the apoptotic markers, cleaved-caspase3 and BCL2 were determined by Western blot on day 28 to verify the above finding (Figure 2C). It was found that cleaved-caspase3 expression was decreased in the BMSC group by comparison with the SCI group (*p* < 0.001) and BMSC-GW group (*p* < 0.01), as well as reduced in the BMSC-Exo group versus the SCI group (*p* < 0.001) (Figure 2D). Nevertheless, BCL2 expression exhibited an opposite trend among groups (most *p* < 0.05), except for no change between the BMSC group and the BMSC-GW group (Figure 2E).

The IF staining was performed to detect neuronal viability in SCI rats on day 28 (Figure 2F). It was discovered that NeuN^+^ cells were raised in the BMSC group versus the SCI group (*p* < 0.001) and the BMSC-GW group (*p* < 0.01). Meanwhile, NeuN^+^ cells were also enlarged in the BMSC-Exo group versus the SCI group (*p* < 0.01) (Figure 2G).

### BMSC-exo inhibited microglia M1 polarization-mediated inflammation in SCI rats

**FIGURE 3:**
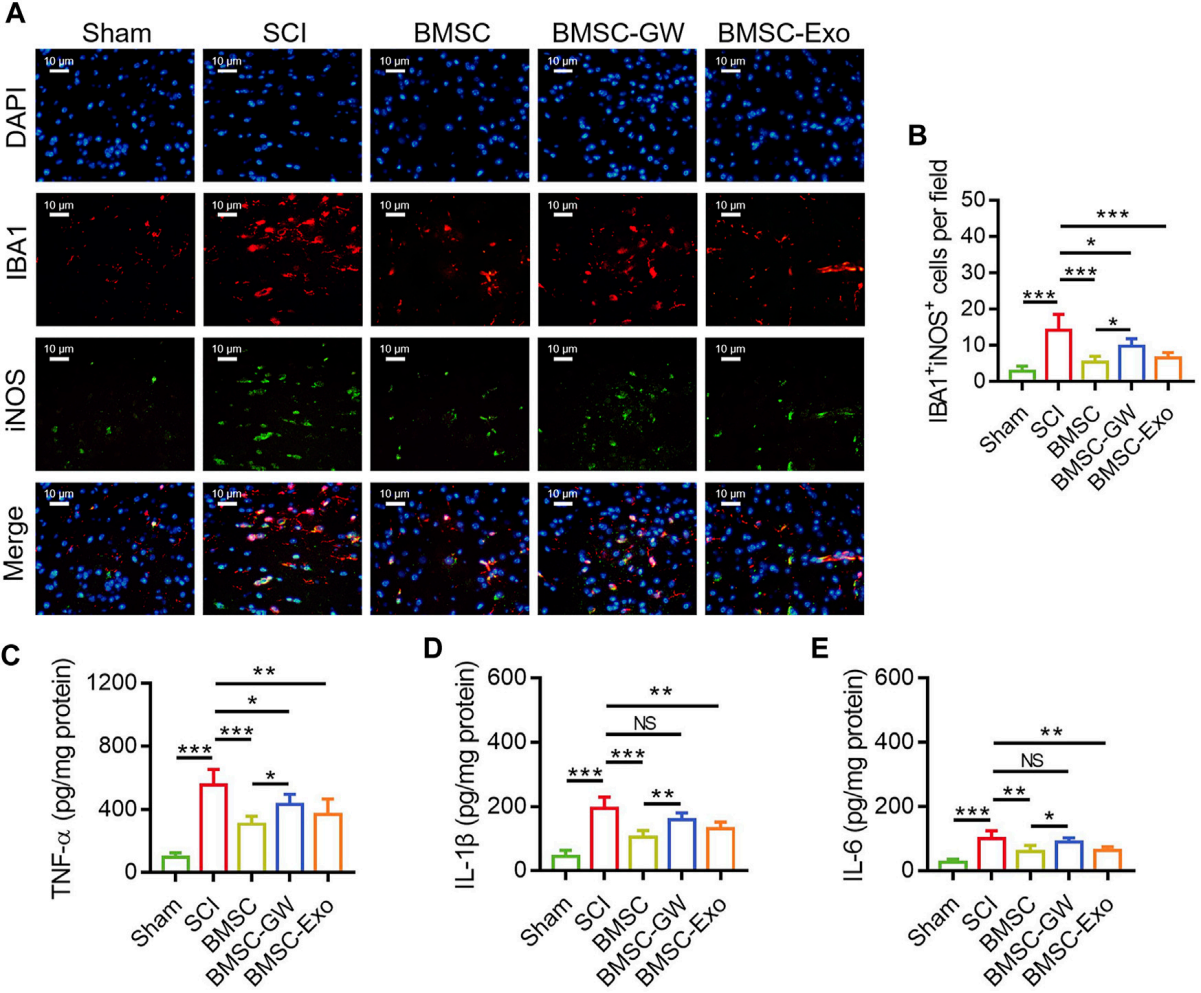
Effect of BMSC-Exo on microglia M1 polarization-mediated inflammation in SCI rats. Representative images of IBA1^+^iNOS^+^ cells by IF staining **(A)**, comparison of IBA1^+^iNOS^+^ cells **(B)**, TNF-α **(C)**, IL-1β **(D)**, and IL-6 **(E)**, among the sham, SCI, BMSC, BMSC-GW, and BMSC-Exo groups. Six biological replicative samples were tested in each group, and 30 rats were tested in total among the sham, SCI, BMSC, BMSC-GW, and BMSC-Exo groups.

The IF staining was performed to detect microglia M1 polarization in SCI rats on day 28 (Figure 3A). It was found that IBA1^+^iNOS^+^ cells were diminished in the BMSC group versus the SCI group (*p* < 0.001) and the BMSC-GW group (*p* < 0.05). Additionally, IBA1^+^iNOS^+^ cells were also reduced in the BMSC-Exo group versus the SCI group (*p* < 0.001) (Figure 3B).

Regarding the inflammatory cytokines, TNF-α (Figure 3C), IL-1β (Figure 3D), and IL-6 (Figure 3E) in the lesions were all decreased on day 28 in the BMSC group by comparison with the SCI group (all *p* < 0.01) and the BMSC-GW group (all *p* < 0.05), as well as diminished in the BMSC-Exo group versus the SCI group (all *p* < 0.01).

### BMSC-exo-derived miR-216a-5p strengthened locomotor performance in SCI rats

**FIGURE 4:**
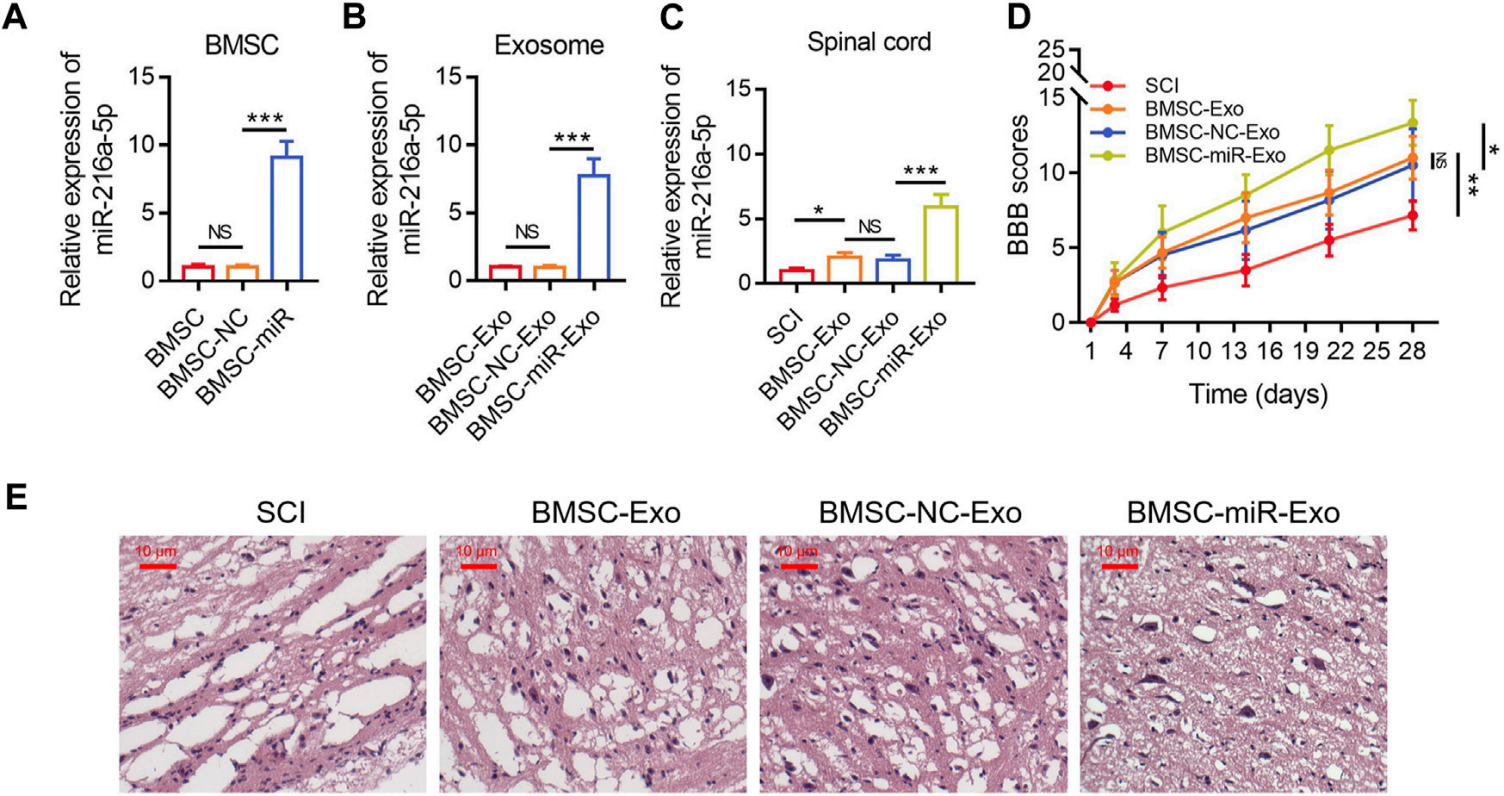
Effect of BMSC-Exo-derived miR-216a-5p on locomotor performance in SCI rats. Comparison of relative miR-216a-5p expression among BMSC, BMSC-NC, and BMSC-miR groups; 3 biological replicative samples were tested in each group **(A)**. Comparison of relative miR-216a-5p expression among BMSC-Exo, BMSC-NC-Exo, and BMSC-miR-Exo groups; 3 biological replicative samples were tested in each group **(B)**. Comparison of relative miR-216a-5p expression **(C)** and BBB score **(D)**, representative images of HE staining **(E)**, among the SCI, BMSC-Exo, BMSC-NC-Exo, and BMSC-miR-Exo groups; 6 biological replicative samples were tested in each group, and 24 rats were tested in total among the SCI, BMSC-Exo, BMSC-NC-Exo, and BMSC-miR-Exo groups.

The relative miR-216a-5p expression in BMSCs was increased following the transfection with miR-216a-5p mimics versus the NC mimics (*p* < 0.001) (Figure 4A), which indicated the transfection was successful. After that, the exosomes of transfected BMSCs were isolated. The relative miR-216a-5p expression in BMSC-Exo was upregulated following the transfection with miR-216a-5p mimics versus the NC mimics (*p* < 0.001) (Figure 4B). The relative miR-216a-5p expression in the spinal cord was increased in the BMSC-miR-Exo group versus the BMSC-NC-Exo group (*p* < 0.001) (Figure 4C). In terms of locomotor performance, the BBB score on day 28 was higher in the BMSC-miR-Exo group than in the BMSC-NC-Exo group (*p* < 0.05) (Figure 4D). Results of HE staining were shown in Figure 4E, which suggested that neuronal injury and inflammation infiltration were attenuated in the BMSC-miR-Exo group versus the BMSC-NC-Exo group.

### BMSC-exo-derived miR-216a-5p suppressed neuronal injury in SCI rats

**FIGURE 5:**
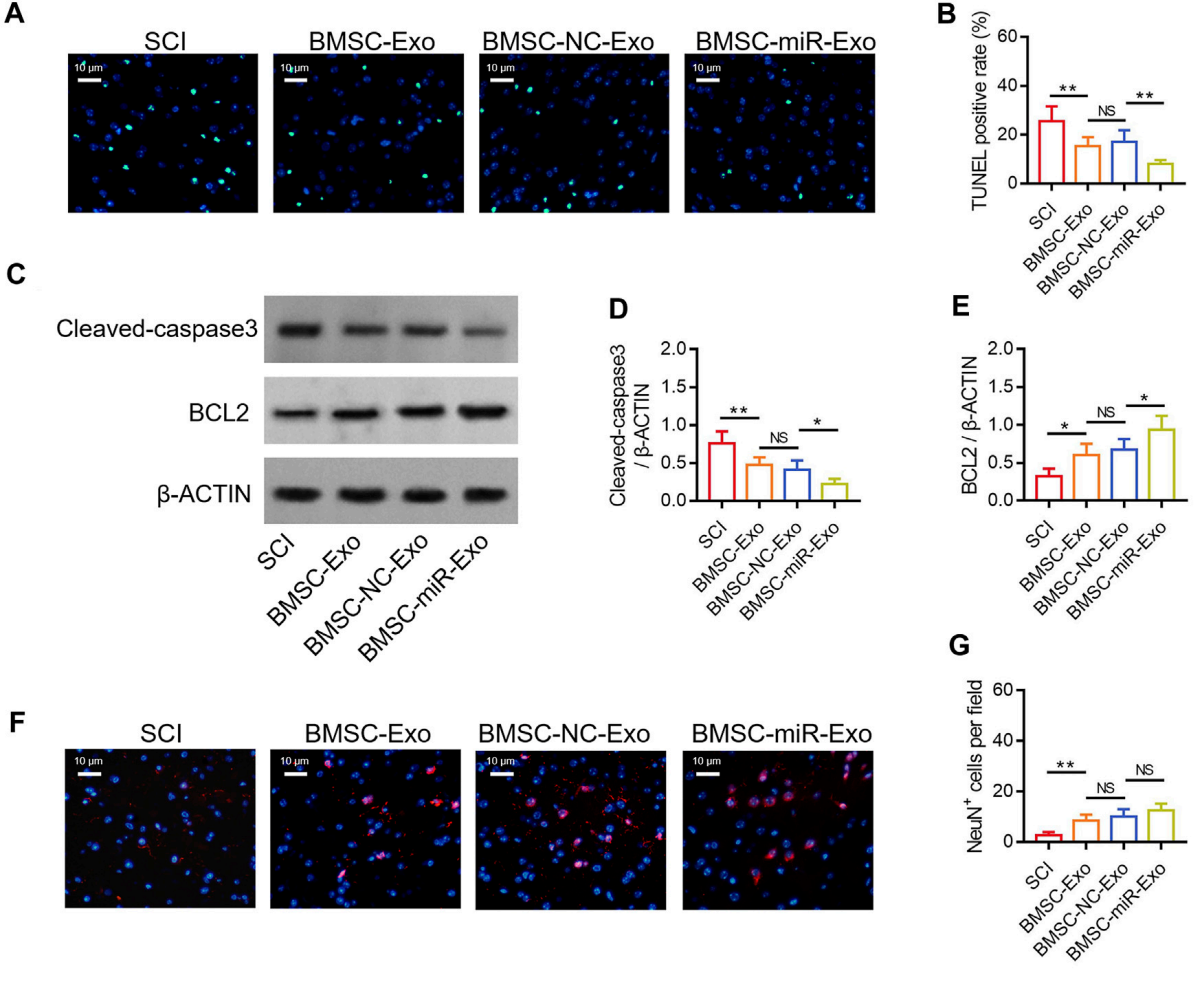
Effect of BMSC-Exo-derived miR-216a-5p on neuronal injury in SCI rats. Representative images of cell apoptosis by TUNEL staining **(A)**, comparison of TUNEL positive rate **(B)**, detection of cleaved-caspase3 and BCL2 by Western blot **(C)**, comparison of cleaved-caspase3 **(D)** and BCL2 **(E)**, representative images of NeuN^+^ cells by IF staining **(F)**, and comparison of NeuN^+^ cells **(G)**, among the SCI, BMSC-Exo, BMSC-NC-Exo, and BMSC-miR-Exo groups. Six biological replicative samples were tested in each group, and 24 rats were tested in total among the SCI, BMSC-Exo, BMSC-NC-Exo, and BMSC-miR-Exo groups.

By TUNEL staining (Figure 5A), it was found that the TUNEL positive rate on day 28 was reduced in the BMSC-miR-Exo group versus the BMSC-NC-Exo group (*p* < 0.01) (Figure 5B). Then Western blot was conducted on day 28 to confirm the above finding (Figure 5C), which displayed that cleaved-caspase3 expression was decreased (*p* < 0.05) (Figure 5D) but BCL2 expression was raised (*p* < 0.05) (Figure 5E) in the BMSC-miR-Exo group versus the BMSC-NC-Exo group. By IF staining (Figure 5F), it was figured out that NeuN^+^ cells on day 28 only showed an increasing trend in the BMSC-miR-Exo group versus the BMSC-NC-Exo group but did not achieve statistical significance (*p* > 0.05) (Figure 5G).

### BMSC-exo-derived miR-126a-5p hindered microglia M1 polarization-mediated inflammation in SCI rats

**FIGURE 6:**
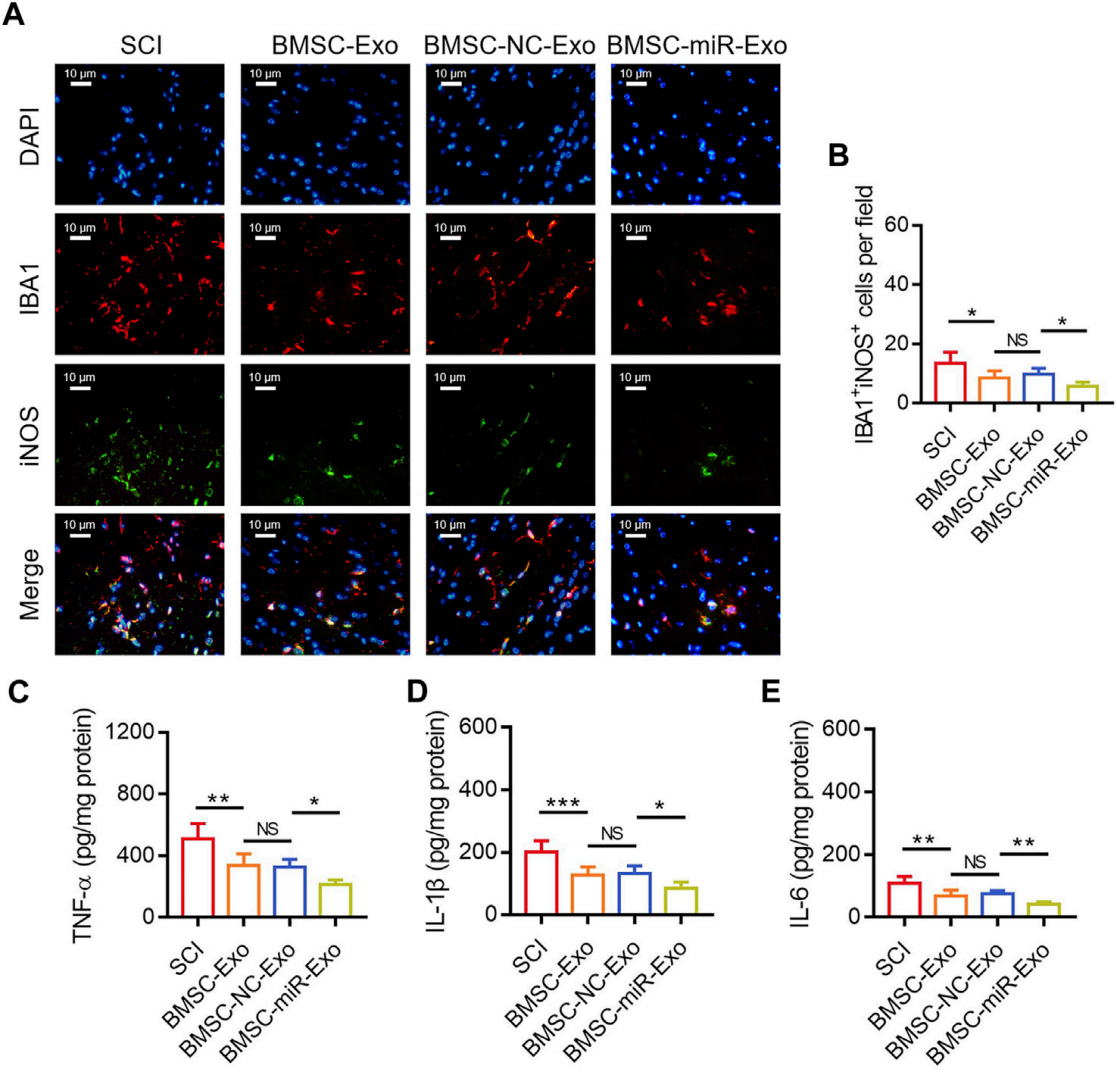
Effect of BMSC-Exo-derived miR-216a-5p on microglia M1 polarization-mediated inflammation in SCI rats. Representative images of IBA1^+^iNOS^+^ cells by IF staining **(A)** and comparison of IBA1^+^iNOS^+^ cells **(B)**, TNF-α **(C)**, IL-1β **(D)**, and IL-6 **(E)**, among the SCI, BMSC-Exo, BMSC-NC-Exo, and BMSC-miR-Exo groups. Six biological replicative samples were tested in each group, and 24 rats were tested in total among the SCI, BMSC-Exo, BMSC-NC-Exo, and BMSC-miR-Exo groups.

By IF staining (Figure 6A), it was discovered that IBA1^+^iNOS^+^ cells on day 28 were reduced in the BMSC-miR-Exo group versus the BMSC-NC-Exo group (*p* < 0.05) (Figure 6B). With respect to the inflammatory cytokines, TNF-α (*p* < 0.05) (Figure 6C), IL-1β (*p* < 0.05) (Figure 6D), and IL-6 (*p* < 0.01) (Figure 6E) in the lesions were all reduced in the BMSC-miR-Exo group versus the BMSC-NC-Exo group.

### BMSC-exo-derived miR-216a-5p suppressed the TLR4/NF-κB pathway

**FIGURE 7:**
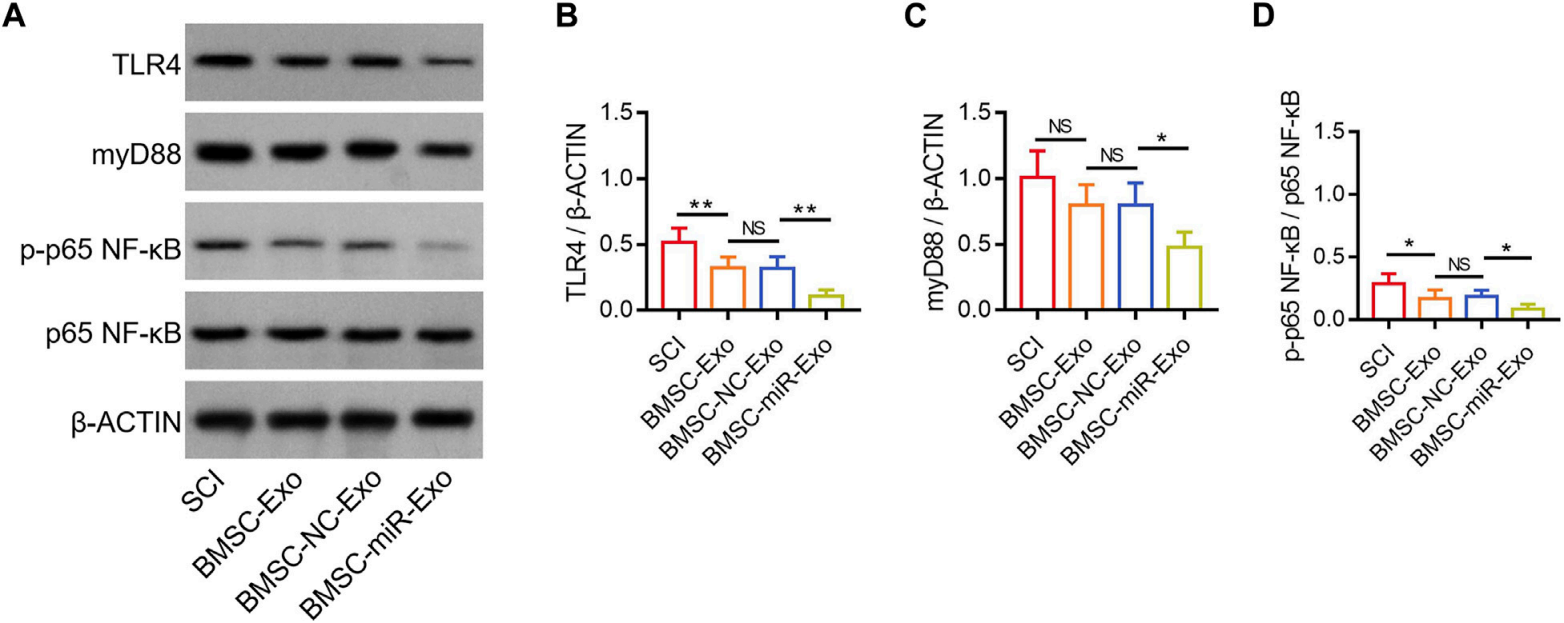
Effect of BMSC-Exo-derived miR-216a-5p on TLR4/NF-κB pathway in SCI rats. Detection of TLR4, myD88, and NF-κB by Western blot analysis **(A)** and comparison of TLR4/β-ACTIN **(B)**, myD88/β-ACTIN **(C)**, and p-p65 NF-κB/p65 NF-κB **(D)**, among the SCI, BMSC-Exo, BMSC-NC-Exo, and BMSC-miR-Exo groups. Six biological replicative samples were tested in each group, and 24 rats were tested in total among the SCI, BMSC-Exo, BMSC-NC-Exo, and BMSC-miR-Exo groups.

According to a previous study, miR-216a-5p inhibits the TLR4/NF-κB pathway to regulate inflammatory response and cell apoptosis (Huang et al., 2023). Notably, another study reports that miR-216a-5p is increased in hypoxic BMSC-Exo, which might potentially participate in the hypoxic BMSC-Exo-mediated microglial polarization by inhibiting the TLR4/NF-κB pathway (Liu et al., 2020). In this study, TLR4, myD88, and p-p65 NF-κB were determined in the lesions by Western blot on day 28 (Figure 7A). It was found that TLR4/β-ACTIN was reduced in the BMSC-miR-Exo group versus the BMSC-NC-Exo group (*p* < 0.01), and in the BMSC-Exo group versus the SCI group (*p* < 0.01) (Figure 7B). However, myD88/β-ACTIN was only decreased in the BMSC-miR-Exo group versus the BMSC-NC-Exo group (*p* < 0.05); although myD88/β-ACTIN showed a trend to decrease in the BMSC-Exo group versus the SCI group, it did not achieve statistical significance (*p* > 0.05) (Figure 7C). In addition, p-p65 NF-κB/p65 NF-κB was decreased in the BMSC-miR-Exo group versus the BMSC-NC-Exo group (*p* < 0.05), as well as in the BMSC-Exo group versus the SCI group (*p* < 0.05) (Figure 7D). These findings suggested that both BMSC-Exo and BMSC-Exo-derived miR-216a-5p inhibited the TLR4/NF-κB pathway.

## Discussion

The therapeutic effect of BMSC-Exo for SCI and the potential underlying mechanisms have been revealed by previous studies (Xin W. et al., 2021; Jia et al., 2021; Sheng et al., 2021). One study finds out that BMSC-Exo improves the function of macrophages by enhancing phagocytosis of myelin debris internalization, which assists in functional recovery after SCI (Sheng et al., 2021). At the same time, a previous study reports that BMSC-Exo preserves the integrity of the blood-spinal cord barrier and enhances the functional recovery after SCI through the tissue inhibitors of matrix metalloproteinase 2/matrix metalloproteinase pathway (Xin W. et al., 2021). In the current study, several interesting findings were discovered. Firstly, BMSC-Exo improved locomotor performance in SCI rats. A possible reason would be that BMSC-Exo might enhance locomotor performance by inhibiting neuronal injury and the microglia M1 polarization-mediated inflammation (Liu et al., 2020; Xu et al., 2020). Secondly, it was found that BMSC-Exo hindered neuronal injury in SCI rats. A reason behind this would be that BMSC-Exo might activate the c-Jun N-terminal kinase 1/c-Jun pathway, endoplasmic reticulum to nucleus signaling 1 (Ern1), etc. to reduce cell apoptosis and improve neuronal cell viability in SCI rats (Li R. et al., 2020; Jiang et al., 2023). Thirdly, BMSC-Exo inhibited microglia M1 polarization-mediated inflammation in SCI rats. This finding was partly in line with a previous study, in which BMSC-Exo ameliorates injury by regulating microglial polarization to inhibit NLRP3 inflammasome-mediated inflammation (Liu X. et al., 2021). Fourthly, it was also discovered that BMSC-Exo inhibited the TLR4/NF-κB pathway in SCI rats. This finding was partly in line with previous studies (Liu et al., 2020; Jiang and Zhang, 2021).

Several studies have concentrated on the protective implication of BMSC-Exo-derived microRNAs in SCI, such as miR-137, miR-29b, miR-124-3p, miR-455-5p, and miR-125a (Yu et al., 2019; Li R. et al., 2020; Chang et al., 2021; Liu et al., 2022; Shao et al., 2023). For instance, one study elucidates that BMSC-Exo-derived miR-137 improves locomotor performance and neuronal cell viability, as well as attenuates tissue injury and inflammation in SCI rats (Shao et al., 2023). At the same time, another study figures out that BMSC-Exo-derived miR-133b preserves neurons, facilitates the regeneration of axons, and enhances hindlimb locomotor performance in SCI rats (Li et al., 2018). Furthermore, BMSC-Exo-derived miR-125a exerts neuroprotective effects in SCI rats by downregulating interferon regulatory factor 5 (IRF5) (Chang et al., 2021). In terms of BMSC-Exo-derived miR-216a-5p, rare studies explore its effect on SCI rats. In this study, it was discovered that BMSC-Exo-derived miR-216a-5p improved locomotor performance, as well as attenuated neuronal injury and microglia M1 polarization-modulated inflammation in SCI rats. The potential reasons would be that: (1) BMSC-Exo-derived miR-216a-5p might regulate IRF5 and phosphatase and tensin homolog (PTEN) to inhibit cell apoptosis (Chang et al., 2021; Zhang et al., 2021). (2) BMSC-Exo-derived miR-216a-5p might mediate PTEN and NF-κB pathways to suppress microglia M1 polarization-induced inflammatory cytokines (Zhang et al., 2021). (3) As discussed above, BMSC-Exo-derived miR-216a-5p could hinder neuronal injury and inflammation in various ways; thereby, it could improve locomotor performance (Chang et al., 2021; Jiang and Zhang, 2021; Wang et al., 2021; Zhang et al., 2021; He et al., 2022). Notably, it should be mentioned that BMSC-Exo-derived miR-216a-5p had the potential to reduce neuronal apoptosis after SCI; meanwhile, BMSC-Exo-derived miR-216a-5p showed a trend to elevate the neuronal viability, but did not achieve statistical significance. Our findings indicated that BMSC-Exo-derived miR-216a-5p might help to attenuate neuronal injury after SCI; however, whether BMSC-Exo-derived miR-216a-5p could promote neuronal regeneration after SCI should be validated by further experiments.

MiR-216a-5p/TLR4/NF-κB pathway participates in cell apoptosis and inflammation in various neurological disorders (Ouyang et al., 2022; Huang et al., 2023). For instance, one previous study reports that miR-216a-5p targets TLR4/NF-κB pathway to inhibit inflammation and the apoptosis of chondrocytes (Huang et al., 2023). Meanwhile, another study indicates that miR-216a-5p directly hinders the TLR4/NF-κB pathway to prevent intestinal epithelial barrier dysfunction (Ouyang et al., 2022). Partly in line with these previous studies (Ouyang et al., 2022; Huang et al., 2023), the present study also figured out that BMSC-Exo-derived miR-216a-5p inhibited the TLR4/NF-κB pathway in SCI rats. According to previous studies, miR-216a-5p could directly target TLR4, thereby hindering the subsequent activation of NF-κB (Ouyang et al., 2022; Huang et al., 2023). Therefore, it was speculated that the inhibition of miR-216a-5p on neuronal injury and inflammation was mediated by preventing the TLR4/NF-κB pathway in SCI rats. However, this speculation needed further experiments to validate. It should be clarified that we applied p-p65/p65 to reflect the activation or inhibition of the NF-κB pathway, which was in line with previous studies (Wang et al., 2019; Yang et al., 2020). The reason for detecting p-p65 was as follows: p65 was a member of the NF-κB pathway, which exerted biological function by being phosphorylation, and p-p65 was the phosphorylated form of p65; thus, the change of p-p65/p65 could reflect the activation or inactivation of the NF-κB pathway. In the current study, it was found that p-p65/p65 was decreased by BMSC-Exo-derived miR-216a-5p, which indicated that BMSC-Exo-derived miR-216a-5p inactivated the NF-κB pathway. In terms of the detection of TLR4/β-ACTIN and myD88/β-ACTIN, we used β-ACTIN as the internal reference to reflect the expression of TLR4 and myD88 proteins, which was in line with previous studies (Liu et al., 2020; Rong et al., 2021; Gu et al., 2023). Overall, this study discovered that BMSC-Exo-derived miR-216a-5p reduced the TLR4/β-ACTIN, myD88/β-ACTIN, and p-p65/p65 in SCI rats, which indicated that BMSC-Exo-derived miR-216a-5p inactivated the TLR4/NF-κB pathway in SCI rats.

In summary, BMSC-Exo-derived miR-216a-5p improves functional recovery by attenuating neuronal injury and microglia-mediated inflammation in SCI, which may benefit from its inactivation of the TLR4/NF-κB pathway. However, more shreds of evidence are required to validate our findings.

## Data Availability

The original contributions presented in the study are included in the article/Supplementary Material, further inquiries can be directed to the corresponding author.
