# Supplementary figures and images for: Bone marrow mesenchymal stem cell exosomes-derived microRNA-216a-5p on locomotor performance, neuronal injury, and microglia inflammation in spinal cord injury
Source: Front Cell Dev Biol. 2023 Sep 12;11:1227440. doi: 10.3389/fcell.2023.1227440 (PMC10520706; doi:10.3389/fcell.2023.1227440)

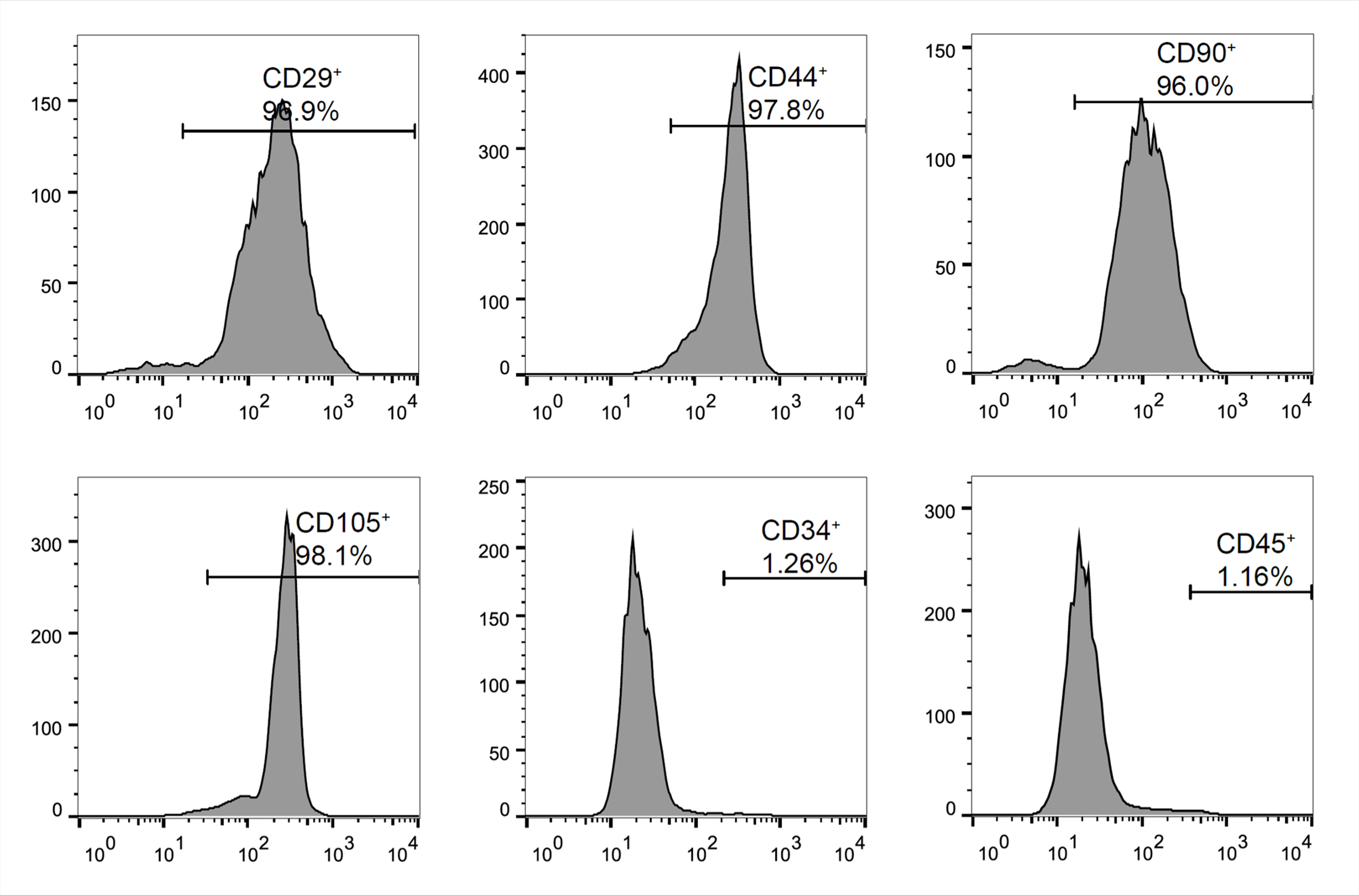

Supplement: Supplementary file 1 [file Image1.TIF]
